# Low-Temperature Fluoro-Borosilicate Glass for Controllable Nano-Crystallization in Glass Ceramic Fibers

**DOI:** 10.3390/nano13101586

**Published:** 2023-05-09

**Authors:** Qichao Zhao, Jianfeng Li, Tingyu Zha, Penghui Zhang, Yi Long, Zaijin Fang

**Affiliations:** Guangdong Provincial Key Laboratory of Optical Fiber Sensing and Communications, Institute of Photonics Technology, Jinan University, Guangzhou 511443, Chinazz2419917512@stu2021.jnu.edu.cn (T.Z.); phzhang@stu2022.jnu.edu.cn (P.Z.);

**Keywords:** glass ceramic, nano-crystallization, glass, luminescence, optical fiber

## Abstract

A fluorosilicate (FS) nano-crystallized glass ceramic (NGC) is one of the most commonly used gain materials for applications in optical devices due to its excellent thermal stability as well as high-efficiency luminescence. However, FS glass can hardly be used to prepare NGC fibers due to its high preparation temperature. Here, a series of low-temperature fluoro-borosilicate (FBS) glasses were designed for the fabrication of active NGC fibers. By modulating B_2_O_3_, the preparation temperature of FBS glass was reduced to 1050 °C, and the crystallization in FBS NGCs was more controllable than in FS NGC. The crystallization of the impure phase was inhibited, and single-phase rare earth (RE)-fluoride nanocrystals were controllably precipitated in the FBS NGCs. The 40Si-20B FBS NGC not only exhibited a higher optical transmittance, but the luminescence efficiency was also much higher than traditional FS NGCs. More importantly, NGC fibers were successfully fabricated by using the designed FBS glass as core glass. Nanocrystals were controllably precipitated and greatly enhanced, and upconversion luminescence was observed in NGC fibers. The designed FBS NGCs provided high-quality optical gain materials and offered opportunities for fabricating a wide range of NGC fibers for multiple future applications, including fiber lasers and sensors.

## 1. Introduction

Actively optical fibers have been extensively investigated owing to their potential for diverse applications in fiber lasers, fiber amplifiers and fiber sensors [1,2,3]. Generally, the properties of optical fibers are deeply governed by glass matrices. The past few decades have witnessed developments in optical gain glasses for exploiting optical fibers featuring high-efficiency and multiple-wavelength luminescence. Nano-crystallized glass ceramic (NGC), a significant composite containing a large number of glass phases as well as specific nanocrystals, has been developed as desirable optical gain material because it possesses the advantage of glass featuring high optical transmittance and that of crystal, exhibiting excellent coordinated environments for efficient luminescence [4,5,6,7,8,9,10]. Fluorosilicate (FS) NGC has been developed as one of the most common optical gain materials because it possesses strong framework structures constructed by [SiO_4_] networks as well as efficient luminescence when active ions are incorporated into fluoride crystals featuring intrinsically low phonon energy. Thus, FS NGC exhibited excellent thermal stability as well as high-efficiency luminescence. So far, a variety of FS NGCs containing NaYF_4_, LaF_3_, YF_3_, KSc_2_F_7_ and KYb_3_F_10_ have been designed to enhance the luminescence efficiency of active ions [11,12,13,14,15,16,17,18,19,20].

More importantly, FS NGC maintains the unique fiber drawing properties of glass when it is heated to soften with temperature. This provides significant opportunities for the fabrication of optical fibers containing nanocrystals based on NGCs. Since nanocrystals are precipitated from a glass matrix in NGC fibers, the luminescence efficiency is greatly enhanced, and the emission wavelength is modulated when active ions are successfully incorporated into crystal lattices, providing highly promising developments for efficient fiber devices, such as high-power fiber lasers, broadband fiber amplifiers and fiber sensors [21,22,23]. So far, NGC fibers containing functional nanocrystals have been fabricated via various techniques [24,25,26,27]. To achieve high-quality NGC fibers, the key point is the controllable precipitation of nanocrystals in fibers. Recently, the “melt-in-tube” technique has been proven to be the most feasible method for the fabrication of NGC fibers [28,29,30,31,32]. In the fiber drawing process, fibers are a precursor at the drawing temperature where the fiber core glass is melted while the clad is softened. The traditional FS of glass is not suitable for the fabrication of NGC fibers due to high preparation temperatures (>1450 °C), which lead to the heavy volatilization of fluorine in the glass melt and a drastic interfacial reaction between the fiber core and clad during the melt-in-tube fiber drawing process. Crystal is not controllably precipitated in the fiber, and the fabrication of NGC fiber is unsuccessful. These shortcomings limit the practical applications of FS NGCs in fiber devices. Therefore, it is necessary to explore novel FS glass networks for the fabrication of high-quality NGCs and NGC fibers.

Generally, fluoro-borosilicate (FBS) glass, exhibiting a lower preparation temperature by adding B_2_O_3_ into the FS glass, has been developed as an optical gain material [33]. Moreover, fluoride nanocrystals can also be precipitated from FBS glass to form efficiently luminescent NGCs [34,35]. In this work, a system of novel fluoro-borosilicate (FBS) glass networks was designed to prepare active NGCs and NGC fibers embedded with fluoride nanocrystals and achieve high-efficiency luminescence. The incorporation of B_2_O_3_ into FS glass significantly reduced the preparation temperature, decreased the volatilization of fluorine and decreased the reaction between the fiber core and cladding. Additionally, B-O bonds decreased the aggregation of fluoride glass networks in FS glass and thus obtained controllable crystallization in NGCs [36]. The prepared NGCs provided a high-quality gain material for efficient luminescence and offered significant opportunities for the fabrication of active NGC fibers.

## 2. Materials and Methods

### 2.1. Materials Preparation

In this work, glasses with the nominal components of (60-x)SiO_2_-xB_2_O_3_-20KF-20ZnF_2_ were chosen as the host glasses (in mol %) (x = 0, 5, 10, 20, 30 and 40). These samples are referred to as 60Si-FS, 50Si-10B, 40Si-20B, 30Si-30B and 20Si-40B FBS glasses, respectively. In addition, YbF_3_ and ErF_3_ were doped into the glasses to achieve upconversion (UC) luminescence. All glasses were prepared using the melt-quenching method. A stoichiometric mixture of 30 g of the reagent grades SiO_2_ (99.99%), ZnF_2_ (99.99%), KF (99.99%), YbF_3_ (99.99%) and ErF_3_ (99.99%) was mixed thoroughly and then melted in a platinum-rhodium crucible at temperatures ranging from 1000 to 1450 °C. The crucible was covered during the glass preparation process. The glass melt was poured onto a cold brass mold and pressed with another brass plate to prepare the precursor glasses. Then, the precursor glasses were heat treated to fabricate NGCs. In order to prepare NGC fibers, the Yb^3+^-Er^3+^ co-doped 40Si-20B FBS glass and commercial borosilicate glass (composition: 80SiO_2_-11B_2_O_3_-9Na_2_O (mol%)) were selected as the core and clad material, respectively. The NGC fibers were fabricated via the melt-in-tube method [26]. The precursor fiber was drawn at 1080 °C, where the core glass melted while the clad glass was softened. By quick drawing (15 m/s), the FBS precursor fiber was prepared. Then, the precursor fibers were treated with heat to prepare NGC fibers.

### 2.2. Characterizations

Differential scanning calorimetry (DSC, STA 449 C, NETZSCH, Bavaria, Germany) analysis was performed in an argon atmosphere to study the glass transition temperature and crystallization temperature at a rate of 10 K min^−1^. To identify the crystalline phase in NGCs, X-ray diffraction (XRD) patterns were performed on an X-ray diffractometer (Bruker, Fällanden, Switzerland) using Cu/Ka (λ = 0.1541 nm) radiation. The morphology and size distribution of the nanocrystals in NGCs were measured by high-resolution transmission electron microscopy (HR-TEM) (Tecnai G2, FEI, Omaha, NE, USA). The UC emission spectra of the samples were recorded using an Edinburgh FLS980 fluorescence spectrometer (Edinburgh Instruments, Edinburgh, UK). Transmission spectra were measured by a UV/VIS/NIR spectrophotometer (Lambda-900, PerkinElmer, Waltham, MA, USA). A 980 nm laser diode (LD) was used as the excitation source to measure the UC emission spectra. Quantum yields of the samples were measured using the same spectrometer equipped with an integrating sphere. The measurement details of the quantum yield values are similar to our previous works [10]. All measurements were carried out at room temperature.

## 3. Results and Discussion

**Figure 1:**
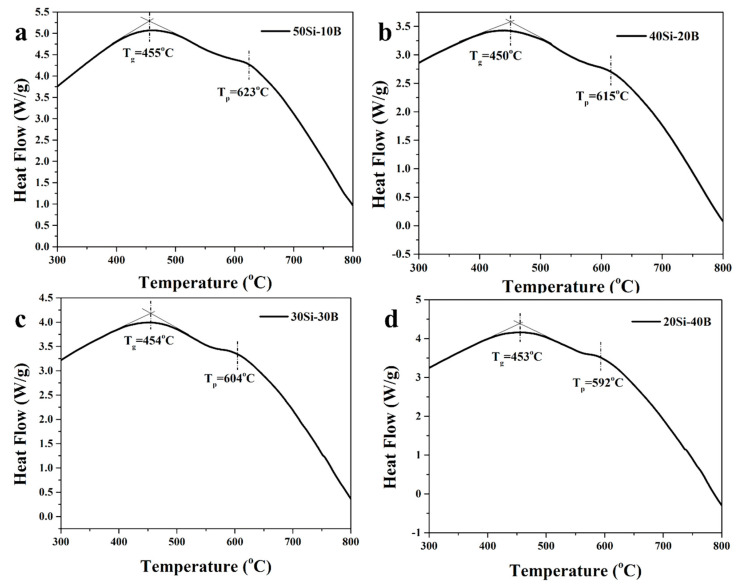
DSC curves of 50Si-10B (**a**), 40Si-20B (**b**), 30Si-30B (**c**) and 20Si-40B (**d**) FBS glasses.

The DSC curves of the FBS glasses are shown in Figure 1. From the DSC curve, the glass transition temperature (T_g_) of 50Si-10B, 40Si-20B, 30Si-30B and 20Si-40B glass was found to be 455, 450, 454 and 453 °C, respectively. The crystallization peak temperature (T_p_) of 50Si-10B, 40Si-20B, 30Si-30B and 20Si-40B glass was found to be 623, 615, 604 and 592 °C, respectively. To prepare high-quality NGCs containing fluoride nanocrystals, the heat treatment temperature for the glasses was set between the T_g_ and T_p_.

**Figure 2:**
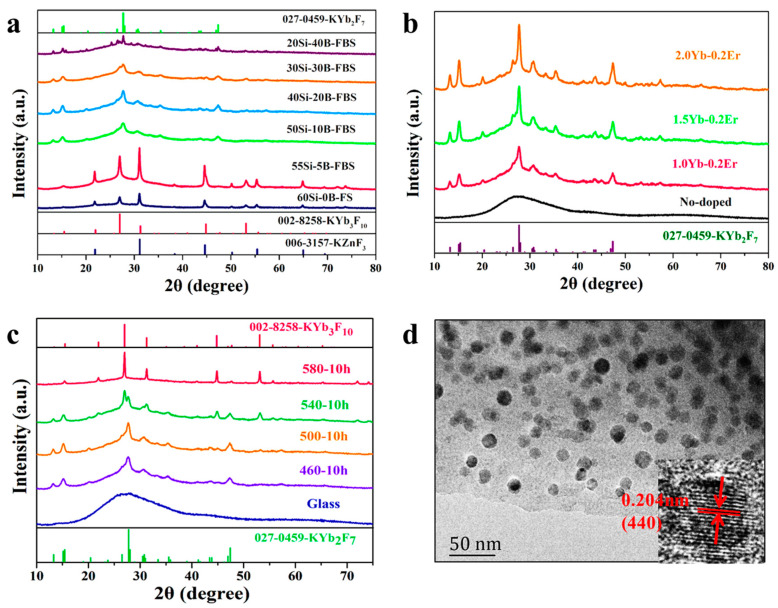
(**a**) Compared XRD patterns of 1.0Yb^3+^−0.2Er^3+^ co-doped FS and FBS NGCs when heat treated at 520 °C for 10 h, (**b**) XRD patterns of no-doped and xYb^3+^−0.2Er^3+^ co-doped 40Si-20B NGCs (x = 1.0–2.0) when heat treated at 520 °C for 10 h, (**c**) XRD patterns of 1.0Yb^3+^−0.2Er^3+^ co-doped 40Si-20B glass and NGCs when heated at different temperatures, (**d**) TEM image of 1.0Yb^3+^−0.2Er^3+^ co-doped 40Si-20B NGC when heated at 580 °C for 10 h; the inset is the enlarged HR-TEM image.

To reveal the evolution of crystalline phases in the NGCs, the XRD patterns of Yb^3+^-Er^3+^ co-doped FS and FBS NGCs were carefully investigated and are shown in Figure 2a. It is clear that KYb_3_F_10_ crystals were precipitated in the 60Si-0B FS NGC. Meanwhile, the diffraction peaks of the KZnF_3_ crystal were also observed in the XRD pattern of 60Si FS NGC. KYb_3_F_10_ crystals confined Yb^3+^ ions in fluoride crystal environments featuring low phonon energy [10,17], while KZnF_3_ provided no appropriate lattice for the incorporation of RE ions. Thus, the crystallization of KZnF_3_ in the FS NGC made no contribution to the enhancement of luminescence for RE, ions and KZnF_3_ was an impure phase in the FS NGC. For the 55Si-5B FBS NGC, KZnF_3_ and KYb_3_F_10_ crystals were also both precipitated from the glass via heat treatment. The intensities of the diffraction peaks in 55Si-5B FBS NGC were all higher than the corresponding peaks in 60Si FS NGC, indicating that more KZnF_3_ and KYb_3_F_10_ crystals were precipitated in 55Si-5B FBS NGC. However, the diffraction peak of KZnF_3_ crystals was not observed in the XRD patterns of FBS NGCs when the B_2_O_3_ concentration increased from 10 to 40%. More importantly, only the diffraction peaks of KYb_2_F_7_ crystals were precipitated in XRD patterns of 50Si-10B, 40Si-20B and 30Si-30B NGCs, proving that pure RE-fluoride crystals were precipitated in these FBS NGCs. For 20Si-40B NGC, the diffraction peaks of KYb_2_F_7_ crystals were weak, and other impure phases could be observed in the XRD patterns. Therefore, the crystallization of the impure KZnF_3_ phase was successfully inhibited, and pure RE-fluoride crystals were controllably precipitated in the FBS NGCs with adjustable compositions when B_2_O_3_ concentration, which changed from 10 to 30 mol%.

In order to illuminate the crystallization mechanism in FBS NGCs, the XRD patterns of 40Si-20B FBS NGCs with various concentrations of the dopants are presented in Figure 2b. The XRD pattern of the no-doped sample exhibited broadband, and no feature of the crystal can be found, indicating that no crystal was precipitated in the no-doped sample even though the glass was also heat treated at 520 °C. Interestingly, sharp diffraction peaks of the crystals were observed in the XRD patterns of Yb^3+^-Er^3+^ co-doped NGCs. These peaks all matched well with those of the JCPDS Card of KYb_2_F_7_ (027-0457) crystal, proving that pure KYb_2_F_7_ crystals were precipitated in the 1.0Yb^3+^-Er^3+^ co-doped NGC. Furthermore, the intensities of diffraction peaks for KYb_2_F_7_ crystals were enhanced monotonously when the concentration of Yb^3+^ increased from 1.0 to 2.0 mol%. These results indicate that the crystallization in the FBS NGC was highly dependent on the doping of Yb^3+^. This crystal was not precipitated from the no-doped host glass, and a small number of dopants induced the crystallization in NGCs. This is called dopant-induced crystallization [10].

In the 60Si-FS glasses, interpenetrating phase separation was observed in the networks [37]. Yb^3+^ ions were distributed in the separated fluoride networks and worked as a crystallization center to induce the precipitation of KYb_3_F_10_ crystals (Figure 2a) [10]. Moreover, a large number of ZnF_2_ and KF were distributed in the fluoride networks and thus, KZnF_3_ crystals were precipitated in the FS NGC. In the FBS glasses, [BO_3_], units were distributed among the glass networks and worked as network modifiers when the content of B_2_O_3_ was low. A part of the Si-O frameworks was broken, and the local viscosity was reduced when 5 mol% B_2_O_3_ was incorporated into the glass. Thus, more KYb_3_F_10_ and KZnF_3_ crystals were precipitated in the 55Si-5B FBS NGC after the heat treatments compared to 60Si NGC (Figure 2a). However, when the content of B_2_O_3_ in the FBS glass increased to 10 mol%, a “boron anomaly” occurred in FBS glasses. [BO_4_] tetrahedrons appeared in the glass networks, worked as network formers and constructed the framework structure of glass together with [SiO_4_] tetrahedrons. The micro phase-separation also occurred in FBS glass networks, and thus, RE-fluoride crystals were precipitated from the separated fluoride networks, and crystallizations were induced by the doping of Yb^3+^ in the FBS NGCs (Figure 2a,b), which was similar to that of FS NGC. Actually, two molar [BO_4_] units were produced when one molar B_2_O_3_ replaced one SiO_2_. Thus, the framework structures of glass were strengthened, and fluoride networks were dispersed by the introduction of B_2_O_3_ [36], which restrained the precipitation of the impure KZnF_3_ phase, as presented in Figure 2a,b. Therefore, the design of FBS NGCs inhibited the precipitation of the impure phase and RE-fluoride crystals were controllably precipitated in a system of FBS NGCs when the glass composition varied in a large range.

Figure 2c shows the XRD patterns of the 40Si-20B glass and NGCs. Owing to the amorphous morphology of the glass, a broad band was observed in the XRD pattern of the glass, indicating that no crystals were precipitated in the precursor glass. Only the diffraction peaks of KYb_2_F_7_ crystal were observed in the XRD patterns of samples heat treated at 460 and 500 °C for 10 h. The diffraction peaks of KYb_3_F_10_ were also observed in the XRD pattern apart from those of KYb_2_F_7_ when the sample was heat treated at 540 °C for 10 h. However, only the peaks of KYb_3_F_10_ crystals could be observed in the XRD patterns of NGC when heat treated at 580 °C for 10 h. The intense diffraction peaks indicate that a large number of KYb_3_F_10_ crystals were precipitated in NGCs. These results prove that the crystalline phases in the FBS NGCs were also modulated by heat treatments and transfer from KYb_2_F_7_ to KYb_3_F_10_ crystals when the temperature increased from 460 to 580 °C.

Additionally, the TEM image of 1.0Yb^3+^-0.2Er^3+^ co-doped 40Si-20B NGC heat treated at 580 °C for 10 h, as shown in Figure 2d, revealed that crystal particles were uniformly dispersed in the glass matrix with a diameter from 5 to 30 nm. The interval of crystal lattice fringes could be measured directly in the HR-TEM image in the inset of Figure 2d, and its value was about 0.204 nm, which corresponded to the (440) crystal facet of KYb_3_F_10_, proving the precipitation of KYb_3_F_10_ nanocrystals in the 40Si-20B NGC.

**Figure 3:**
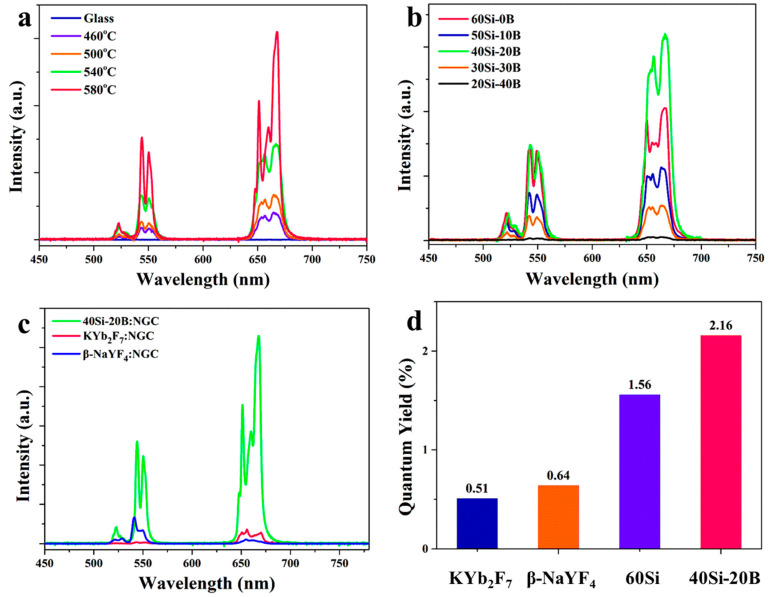
(**a**) Emission spectra of 1.0Yb^3+^−0.2Er^3+^ co-doped 40Si-20B glass and NGCs, (**b**) Emission spectra of 1.0 Yb^3+^−0.2Er^3+^ co-doped FS and FBS NGCs, (**c**) Compared emission spectra and (**d**) Quantum yield values of different NGCs.

Owing to high phonon energy in the FBS glass networks, no emission was observed in the 40Si-20B glass, as shown in Figure 3a. As proved above, KYb_2_F_7_ and KYb_3_F_10_ crystals were controllably precipitated in the FBS NGCs. Owing to the precipitation of RE-fluoride crystals, RE ions are incorporated into the crystal structure from the glass networks during the heat treatments. The RE-fluoride crystals possessed lower phonon energy compared to the glass networks, resulting in the high emission efficiency of RE ions in the NGCs. As shown in Figure 3a, intense emission peaks attributed to UC emissions of Yb^3+^-Er^3+^ ion pairs were observed in the NGCs. The peaks around 522, 540 and 650 nm were assigned to ^2^H_11/2_⇒^4^I_15/2_, ^4^S_3/2_⇒^4^I_15/2_, and ^4^F_9/2_⇒^4^I_15/2_ transitions of Er^3+^ ions, respectively. The UC emission intensity of NGCs was enhanced monotonously when the heat treatment temperature increased from 460 to 580 °C. The phonon energy of the KYb_3_F_10_ crystal was as low as 387 cm^−1^. The emission intensity of NGC heat treated at 580 °C increased dramatically because a large number of KYb_3_F_10_ crystals were precipitated in the NGC. Accordingly, the controllable crystallization in NGCs significantly enhanced the UC emissions of Yb^3+^-Er^3+^ pairs.

The compared emission spectra of FS and FBS NGCs when heat treated at 520 °C for 10 h are shown in Figure 3b. Excited by the use of a 980 nm laser diode, the emission intensity was enhanced by the addition of B_2_O_3_ into NGCs, reaching a maximum of 20 mol% B_2_O_3_. The emission intensity decreased obviously when the B_2_O_3_ concentration increased to 30%, and the emission in 20Si-40B NGC was very weak due to a small number of RE-fluoride crystals in the NGC. The emission intensity in 40Si-20B FBS NGC was even higher than in 60Si FS NGC. The incorporation of B_2_O_3_ into the glass enhanced the UC emission intensity of Er^3+^ in the NGC, and 40Si-20B NGC was the most efficient gain material for UC luminescence.

More importantly, the UC emission intensity of Er^3+^ in the 40Si-20B NGC was much higher than that in traditional β-NaYF_4_ and KYb_2_F_7_ NGC, as shown in Figure 3c. In past studies, the quantum yields of UC luminescence in NGCs were all below 1%. The quantum yield of UC luminescence in 40Si-20B NGC was as high as 2.16%, which is higher than 60Si FS NGC (1.56%) and even 3.4 and 4.2 times higher than that of the traditional β-NaYF_4_ (0.64%) and KYb_2_F_7_ (0.51%) NGC, respectively (Figure 3d). The NGC containing β-NaYF_4_ crystals has been considered one of the most efficient luminescent NGC [38]. RE ions are incorporated into the crystal structures via ionic substitution processes for Y^3+^. Owing to the ionic mismatch between RE and Y^3+^ ions, the quantity of active ions incorporated into crystal structures in β-NaYF_4_ NGC is usually small. For our 40Si-20B FBS NGC, RE-fluoride crystals were controllably precipitated from the glass induced by the doped RE ions. RE ions were all confined in the fluoride crystals during the crystallization process of KYb_3_F_10_ [10], resulting in a more efficient UC emission in 40Si-20B FBS NGC. In order to precipitate KYb_2_F_7_ crystals in the traditional NGC, a large number of YbF_3_ (~6%) was added to the glass [39]. The highly concentrated RE ions in NGCs lead to a heavy absorption of excited light, the probability of non-radiative transitions was very high, and the thermal quenching of luminescence was severe in the traditional KYb_2_F_7_ NGC. However, owing to the dopant-induced crystallization of FBS NGCs, the concentrations of Yb^3+^ were low and thermal quenching of luminescence was slight. Thus, the quantum yield in the traditional KYb_2_F_7_ NGC was much lower than in 40Si-20B NGC. This indicates that dopant-induced crystallization in the FBS NGC made a contribution to the highest efficiency of UC luminescence and provided significant opportunities for overcoming the bottleneck in the UC luminescence efficiency of traditional NGCs.

**Figure 4:**
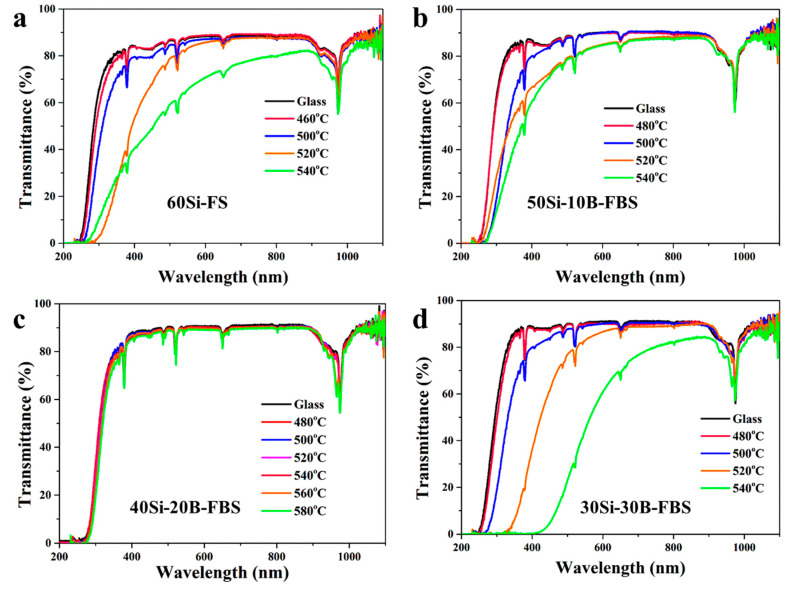
Transmission spectra of (**a**) 60Si, (**b**) 50Si-10B, (**c**) 40Si-20B and (**d**) 40Si-20B glass and NGCs.

The transmission spectra of FS and FBS NGCs are shown in Figure 4. The peaks at 976nm are all attributed to Yb^3+^: ^2^F_7/2_⇒^2^F_5/2_ transitions. The peaks at 800, 647, 537, and 520 nm were ascribed to transitions from the ground state ^4^I_15/2_ to excited levels ^4^I_9/2_, ^4^F_9/2_, ^4^S_3/2_, and ^2^H_11/2_ of Er^3+^, respectively. The transmittances of 60Si FS NGCs were lower than that of the precursor glass. The transmittances were reduced dramatically when the heat treatment temperature was increased. The scattering caused by the crystal particles in 60Si NGC led to a decrease in the transmittance, as shown in the transmission spectrum (Figure 4a). For the 50Si-10B FBS samples, the transmittances of NGCs also reduced when the heat treatment temperature increased. The transmittances of 50Si-10B NGCs were higher than those of 60Si FS NGCs. Interestingly, the transmittances of 40Si-20B NGCs were almost equal to that of the precursor glass. The 40Si-20B NGCs samples were very all transparent, and their transmittance was as high as 90% at 600 nm even though the sample was heat treated at 580 °C for 10 h. As presented in the XRD pattern in Figure 2a, KZnF_3_ crystals were precipitated in the 60Si NGC. The peaks of KZnF_3_ in 60Si NGC were sharper than those of KYb_2_F_7_ crystals in FBS NGCs. The average sizes of KZnF_3_ crystals were larger than that of KYb_2_F_7_ crystals, which resulted in heavy optical scattering and the low optical transmittance of 60Si NGC. The refractive index for the KYb_3_F_10_ crystal was about 1.48, and that of FBS glass was measured at 1.49 at 633 nm. The small difference between the refractive index of KYb_3_F_10_ crystal and the glass matrix made a contribution to the high transmittance of NGCs. Moreover, the transmittances of 30Si-30B NGCs decreased dramatically by the heat treatments due to the heavy separation of B-O phases when too much B_2_O_3_ was added to the glass. Therefore, the incorporation of B_2_O_3_ into FS glass restrained the precipitation of the impure phase (KZnF_3_) and obviously increased the optical transmittance of NGCs. 40Si-20B NGCs exhibited the highest transmittances among the NGCs.

As proved above, the RE ions that doped 40Si-20B NGC exhibited the most efficient UC luminescence and highest optical transmittance. Moreover, the crystallization of RE-fluoride nanocrystals in 40Si-20B NGC was more controllable compared to FS NGC. This indicated that the 40Si-20B NGC was a more desirable gain material for optical devices. Previously, we have tried to fabricate NGC fiber by using 60Si FS glass as the core glass through a melt-in-tube method. However, no nanocrystal was precipitated in the fiber due to the volatilization of fluorine at the high preparation temperature.

The compositions of the FS and FBS glasses were quantitatively established through the use of X-Ray Fluorescence (XRF) measurements (Table 1). These measured compositions differed from the nominal values. Fluorine losses for FS and FBS glasses were calculated at 40.35 and 27.60 mass%, respectively. The preparation temperature of 40Si-20B FBS glass (1050 °C) was much lower than that of 60Si FS glass (1450 °C), which reduced the volatilization of fluorine in optical fibers during the fiber drawing process. Accordingly, 40Si-20B NGC is an excellent candidate for the fabrication of active NGC fibers.

**Figure 5:**
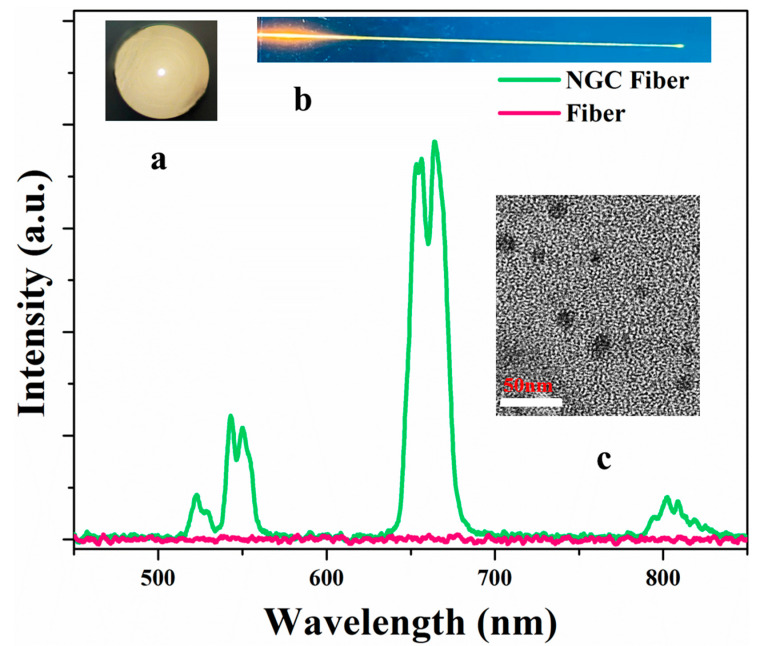
Emission spectra of the precursor fiber and NGC fiber; inset are the photos of the fiber cross section (**a**), NGC fiber irradiated by using a 980 nm laser diode (**b**) and TEM image of NGC fibers (**c**).

The NGC fibers were fabricated by using Yb^3+^-Er^3+^ co-doped 40Si-20B FBS glass as the core glass through a melt-in-tube method. The fiber core exhibited as amorphous and transparent, as shown in the inset Figure 5a. Then, the precursor fibers were heat treated at 580 °C for 10 h to fabricate NGC fibers. Nanocrystals were precipitated in the NGC fibers (inset (Figure 5c)). They were irradiated through the use of a 980 nm laser diode, and an intense yellow emission is observed in the NGC fiber (inset (Figure 5b)), which could be ascribed to the UC emission of Yb^3+^-Er^3+^ pairs as presented in the spectrum in Figure 5. However, no obvious emission was observed in the precursor fiber. These results indicate that the RE-fluoride nanocrystals were controllably precipitated in the fibers. Therefore, the designed FBS glass could provide an excellent matrix when engineering NGC fibers for their application in optical devices.

## 4. Conclusions

In this work, a series of FBS glasses were engineered to fabricate high-quality NGCs and NGC active fibers. The preparation temperature was reduced from 1450 °C to 1050 °C by the addition of B_2_O_3_ into the glasses. Compared to FS NGC, the crystallizations in FBS NGCs were more controlled due to the dispersion of the fluoride network by B_2_O_3_. Impure phase (KZnF_3_) crystals were precipitated in FS NGC, but these were not observed in the XRD patterns of FBS NGCs. Pure phase KYb_2_F_7_ nanocrystals were controllably precipitated in FBS NGCs when the B_2_O_3_ content changed from 10 to 30%. The crystalline phase was modulated from Kyb_2_F_7_ to Kyb_3_F_10_ when the heat treatment temperature increased to 580 °C, and this led to the greatest enhancement of UC emissions in the NGCs. The optical transmittances of FBS NGCs were higher than FS NGC due to their controllable crystallization in FBS NGCs. More importantly, the designed 40Si-20B FBS NGCs exhibited the highest transmittance as well as the most efficient UC luminescence. The NGC fibers were successfully fabricated based on the 40Si-20B FBS glass. A greatly enhanced UC emission was observed in the NGC fibers compared to the precursor fiber. These intriguing properties indicate that the designed FBS NGCs could prove excellent optical gain materials and promising matrices for the manufacturing of optical fiber devices.

## Figures and Tables

**Table 1:** Compositions of the FS and FBS glasses.

	Nominated (Mass %)	Measured (Mass %)	Fluorine Loss (%)
60Si FS glass	18.29	10.91	40.35
40Si-20B FBS glass	16.23	11.75	27.60
